# Evaluation of the efficacy and safety of favipiravir and interferon compared to lopinavir/ritonavir and interferon in moderately ill patients with COVID-19: a structured summary of a study protocol for a randomized controlled trial

**DOI:** 10.1186/s13063-020-04747-8

**Published:** 2020-10-27

**Authors:** Mehdi Hassaniazad, Ali Bazram, Soheil Hassanipour, Mohammad Fathalipour

**Affiliations:** 1 Infectious and Tropical Diseases Research Center, Hormozgan Health Institute, Hormozgan University of Medical Sciences, Bandar Abbas, Iran; 2 Gastrointestinal and Liver Diseases Research Center, Guilan University of Medical Sciences, Rasht, Iran; 3 Department of Pharmacology and Toxicology, Faculty of Pharmacy, Hormozgan University of Medical Sciences, Bandar Abbas, Iran; 4 Endocrinology and Metabolic Research Center, Hormozgan University of Medical Sciences, Bandar Abbas, Iran

**Keywords:** COVID-19, Randomized controlled trial, Protocol, Favipiravir, Lopinavir/ritonavir, Interferon

## Abstract

**Objectives:**

We will evaluate the efficacy and safety of favipiravir and interferon beta-1a compared to lopinavir/ritonavir and interferon beta-1a in patients with confirmed COVID-19, who are moderately ill.

**Trial design:**

This is a phase 3, single-center, randomized, open-label, controlled trial with a parallel-group design carried out at Shahid Mohammadi Hospital, Bandar Abbas, Iran.

**Participants:**

All patients with age ≥ 20 years admitted at the Severe Acute Respiratory Syndrome Departments of the Shahid Mohammadi Hospital, Bandar Abbas, Iran, will be screened for the following criteria.

*Inclusion criteria*:

1. Confirmed diagnosis of infection with SARS-CoV-2 using polymerase chain reaction and/or antibody tests.
2. Moderate COVID-19 pneumonia (via computed tomography and/or X-ray imaging), requiring hospitalization.
3. Hospitalized ≤ 48 h.
4. Signing informed consent and willingness of the participant to accept randomization to any assigned treatment arm.

*Exclusion criteria*:

1. Underlying conditions, including chronic hepatitis, cirrhosis, cholestatic liver diseases, cholecystitis, peptic ulcers, acute and chronic renal failure, and peptic ulcers.
2. Severe and critical COVID-19 pneumonia.
3. History of allergy to favipiravir, lopinavir/ritonavir, and interferon beta-1a.
4. Pregnancy and breastfeeding.

**Intervention and comparator:**

*Intervention group*: favipiravir (Zhejiang Hisun, China) with interferon beta-1a (CinnaGen, Iran). This group will receive 1600 mg favipiravir twice a day for the first day and 600 mg twice a day for the following 4 days with five doses of 44 mcg interferon beta-1a every other day.

*Control group*: lopinavir/ritonavir (Heterd Company, India) with interferon beta-1a (CinnaGen, Iran). This group will receive 200/50 mg lopinavir/ritonavir twice a day for 7 days with five doses of 44 mcg interferon beta-1a every other day.

Other supportive and routine care will be the same in both groups.

**Main outcomes:**

The primary outcome of the trial is the viral load of SARS-CoV-2 in the nasopharyngeal samples assessed by RT-PCR after 7 days of randomization as well as clinical improvement of fever and O_2_ saturation within 7 days of randomization.

The secondary outcomes are the length of hospital stay and the incidence of serious adverse drug reactions within 7 days of randomization.

**Randomization:**

Eligible patients will be allocated to one of the study arms using block randomization in a 1:1 ratio (each block consists of 10 patients). A web-based system will be used to generate random numbers for the allocation sequence. Each number relates to one of the study arms.

**Blinding (masking):**

This is an open-label trial without blinding and placebo control.

**Numbers to be randomized (sample size):**

A total of 60 patients will be randomized into two groups (30 patients in the intervention group and 30 patients in the control group).

**Trial status:**

The trial protocol is version 1.0, 22 July 2020. Recruitment began on 25 July 2020 and is anticipated to be completed by 25 September 2020.

**Trial registration:**

Iranian Registry of Clinical Trials (IRCT) IRCT20200506047323N3. Registered on 22 July 2020.

**Full protocol:**

The full protocol is attached as an additional file, accessible from the *Trials* website (Additional file 1). In the interest in expediting the dissemination of this material, the familiar formatting has been eliminated; this letter serves as a summary of the key elements of the full protocol.

## Supplementary information

**Additional file 1.** Full Study Protocol.

## Data Availability

The corresponding author has access to the final trial information, and the data will be available on reasonable request (contact: M.fathalipour@hums.ac.ir).

