# Supplementary material for: Evaluation of the efficacy and safety of favipiravir and interferon compared to lopinavir/ritonavir and interferon in moderately ill patients with COVID-19: a structured summary of a study protocol for a randomized controlled trial
Source: Trials. 2020 Oct 27;21:886. doi: 10.1186/s13063-020-04747-8 (PMC7588588; doi:10.1186/s13063-020-04747-8)
Supplement: Supplementary file 1 — Additional file 1. Full Study Protocol. [file 13063_2020_4747_MOESM1_ESM.docx]

**Protocol**

This trial protocol has been provided by the authors to give readers additional information about their work.

**Evaluation of the efficacy and safety of Favipiravir and Interferon beta compared to Lopinavir/Ritonavir and Interferon beta in patients with COVID-19: A randomized open-label clinical trial**

Mehdi Hassaniazad ^1^, Ali Bazram ^1^, Soheil Hassanipour ^2^, Mohammad Fathalipour ^3,4^

^1^ *Infectious and Tropical Diseases Research Center, Hormozgan Health Institute, Hormozgan University of Medical Sciences, Bandar Abbas, Iran.*

^2^ *Gastrointestinal and Liver Diseases Research Center, Guilan University of Medical Sciences, Rasht, Iran.*

^3^ *Department of Pharmacology and Toxicology, Faculty of Pharmacy, Hormozgan University of Medical Sciences, Bandar Abbas, Iran.*

^4^ *Endocrinology and Metabolic Research Center, Hormozgan University of Medical Sciences, Bandar Abbas, Iran.*

**Abstract**

An outbreak of severe acute respiratory syndrome coronavirus 2 (SARS-CoV-2) infection and its caused coronavirus disease 2019 (COVID-19) has been reported in China since December 2019. More than 16% of patients developed acute respiratory distress syndrome, and the fatality ratio was about 1-2%. No specific treatment has been reported. Favipiravir as well as Lopinavir/Ritonavir have been used in the treatment of other coronaviruses infection. However, the efficacy of these medications remains controversial. The present study aims to assess the e the efficacy and safety of Favipiravir and Interferon beta compared to Lopinavir/Ritonavir and Interferon beta in patients with covid-19.

A perspective randomized open-label clinical trial will be conducted on 60 hospitalized adult patients with covid-19, who are moderately ill. Patients with positive polymerase chain reaction (PCR) and/or serologic tests will be randomly assigned in a 1:1 ratio to receive, whether 1600 mg Favipiravir twice a day for the first day and 600 mg twice a day for the following four days with five doses of 44 mcg Interferon beta-1a every other day, or 200/50 mg Lopinavir/Ritonavir twice a day for seven days with five doses of 44 mcg Interferon beta-1a every other day.

The primary outcome of the trial is the viral load of SARS-CoV-2 in the nasopharyngeal samples assessed by RT-PCR after seven days of randomization as well as clinical improvement of fever and O2 saturation within seven days of randomization.

The secondary outcomes are the length of hospital stay and the incidence of serious adverse drug reactions within seven days of randomization.

**بررسی اثربخشی و ایمنی ملاتونین در بیماران مبتلا به COVID-19: یک کارآزمایی بالینی تصادفی**

**چکیده**

شيوع عفونت سندرم تنفسي شديد حاد ناشي از کرونا ويروس (SARS-CoV-2) و بيماري کورونا ويروس 2019 (COVID-19) ناشي از آن، از دسامبر سال 2019 در چين گزارش شده است. بيش از 16 درصد از بيماران مبتلا به اين بيماري دچار سندرم شديد تنفسي مي شوند، و حدود 1-2 درصد از اين بيماران دچهر مرگ و مير مي شوند. تاکنون هيچ درماني اختصاصي گزارش نشده است. از فاويپيراوير و همچنين ترکيب لوپيناوير/ريتوناووير در معالجه ساير عفونت هاي کورونا ويروسي استفاده شده است. با اين حال، اثربخشي اين داروها همچنان بحث برانگيز است. مطالعه حاضر با هدف بررسي اثربخشي و ايمني فاويپيراوير و اينترفرون بتا در مقايسه با لوپيناوير/ريتوناوير و اينترفرون بتا در بيماران مبتلا به کوويد 19 انجام خواهد شد. اين کارآزمايي باليني آينده نگر و تصادفي با برچسب باز روي 60 بيمار بزرگسال بستري در بيمارستان مبتلا به کوويد 19 انجام خواهد شد. بيماران با تست واکنش زنجيره اي پليمراز (PCR) مثبت به طور تصادفي به نسبت 1:1 در گروه هاي فاويپيراوير (1600 ميلي گرم دو بار در روز براي روز اول و 600 ميلي گرم سه بار در روز براي 4 روز بعد)، یا 200/50 میلی گرم لوپیناویر / ریتوناویر دو بار در روز به مدت هفت روز با پنج دوز 44 میکروگرم اینترفرون بتا هر روز در میان.

پیامد اولیه آزمایش بار ویروسی SARS-CoV-2 در نمونه های حلق حلقی پس از هفت روز تصادفی سازی توسط RT-PCR و همچنین بهبود بالینی تب و اشباع O2 طی هفت روز از تصادفی سازی خواهد بود.

پیامد ثانویه شامل طول مدت بستری در بیمارستان و بروز واکنش های جدی جانبی دارویی طی هفت روز از تصادفی سازی خواهد بود.

**مقدمه**

بیماری کووید ۱۹ (COVID-19[1]) که به آن بیماری تنفسی حاد ناشی از کرونا ویروس (SARS-CoV-2[2]) نیز گفته می‌شود، بیماری‌ عفونی است که در دسامبر سال 2019، در ووهان چین پدیدار گردید و به سرعت در چین و خارج از آن شیوع یافت (1, 2). به دنبال شیوع این بیماری در سایر نقاط جهان، سازمان بهداشت جهانی[3] در مارس سال 2020 اپیدمی COVID-19 را به عنوان پاندمی اعلام کرد (3).

شایع ترین علائم این بیماری شامل تب، سرفه و تنگی نفس می باشد (4). با این حال، علائم دیگری همچون خستگی مفرط، درد عضلانی، سردرد، ضعف و بی حالی، کاهش حس بویایی و چشایی، اسهال، اسپاسم شکمی، تهوع و استفراغ ممکن است در بیماران بروز پیدا کند (4-6). علائم به طور معمول 5 روز پس از تماس با ویروس شروع می شوند (7). در حالی که اکثر بیماران دچار علائم خفیف می شوند، در برخی موارد این بیماری به پنمونی ویروسی[4] و نارسایی چند اندامی[5] منجر می شود (8). طبق مطالعاتی که اخیرا در چین انجام شده است، حدود 80 درصد از بیماران مبتلا، بیماری خفیف را تجربه می کنند و میزان مرگ و میر حدود 3/2 درصد است. اما در بیماران 70 تا 79 ساله این میزان به 8/14 درصد و در افراد بالای80 سال به 0/18 درصد افزایش می یابد (9). در 2 ژوئیه 2020، حدود 6/10 میلیون مورد ابتلا به این بیماری در بیش از 188 کشور گزارش شده است، که مرگ بیش از 516000 نفر را به همراه داشته است. البته از مبتلایان به این بیماری بیش از 6/5 میلیون نفر بهبودی کامل بدست آورده اند.

بر این اساس، تعداد چشم گیری از ناقلان بدون علامت در جمعیت وجود دارد، و بنابراین احتمالاً میزان مرگ و میر بیش از حد ارزیابی می شود. نهایتاٌ، یک درمان مؤثر برای معالجه بیماران علامت دار در جهت کاهش میزان مرگ و میر به ویژه در گروه های پرخطر، تخفیف علایم بیماری و کاهش احتمال انتقال ویروس در جامعه یک نیاز فوری قلمداد می شود. در حال حاضر تعداد زیادی کار آزمایی بالینی جهت بررسی اثرات داروهای قدیمی ضد ویروس، ضد انگل و ضد باکتری در حال انجام است، زیرا اطلاعات فراوانی در زمینه ایمنی، عوارض جانبی، بیواکی والانسی و تداخلات دارویی این دارو ها در دسترس است. علی رغم تلاش های فراوان در این زمینه تا کنون هیچ واکسن و یا رژیم دارویی در درمان این بیماری توسط سازمان های نظارتی دنیا تائید نشده است (10, 11).

در میان داروهای کاندید برای درمان بیماری COVID-19، استفاده از داروهای ضد ویروس موثر بر سایر بیماری های ویروسی، یک استراتژی جالب است که مورد توجه محققین قرار گرفته است.

فاویپیراویر[6] یکی از جدیدترین RNA پلیمراز است که در بیماری های ویروسی زیادی مورد مطالعه قرار گرفته است و نتایج ضد و نقیضی از این دارو گزارش شده است. در حالی که این دارو در آمریکا و بسیاری از کشور های اروپایی همچنان در درمان آنفولانزا تایید نشده است (12)، در سال 2014، ژاپن فاویپیراویر را برای درمان سویه های آنفلوانزا که نسبت به دارو های ضد ویروس فعلی مقاوم هستند، تصویب کرد. اگرچه اثر بخشی این دارو در درمان بیماری COVID-19 کماکان در دست بررسی می باشد، اما در 15 مارس 2020، این دارو برای درمان Covid-19 در چین تصویب شد (13). در 20 ژوئن 2020، این دارو در هند برای معالجه بیماران Covid-19 تحت نام تجاری Fabiflu تولید شده و مورد تصویب قرار گرفت (14, 15).

از دیگر دارو های ضد ویروسی می توان به ترکیب لوپیناویر/ریتوناویر[7] اشاره کرد که تحت نام تجاری Kaletra فروخته می شود و یک داروی ترکیبی با دوز ثابت برای درمان و پیشگیری از HIV/AIDS[8] است (16). به طور کلی شواهد نشان می دهد، ترکیب لوپیناویر با دوز کم ریتوناویر می تواند در درمان سایر ویروس ها خصوصا رتروویرس ها[9] می شود. این ترکیب در 15 سپتامبر 2000 در اروپا (17) در 19 مارس 2001 توسط سازمان غذا و دارو آمریکا برای درمان HIV/AIDS تصویب شده است (18).

مطالعه حاضر با هدف بررسی اثربخشی و ایمنی داروهای فاویپیراویر و اینترفرون بتا-1a در مقایسه با لوپیناویر/ریتوناویر و اینترفرون بتا-1a در بیماران مبتلا به COVID-19 بستری شده در بیمارستان شهید محمدی شهرستان بندرعباس انجام خواهد شد**.**

**بررسی متون**

علی رغم اینکه تعداد زیادی کارآزمایی بالینی در ازتباط با داروهای مختلف در درمان بیماری COVID-19 انجام شده است، اما کارایی هیچ کدام از این روش های درمانی هنوز به طور قطعی برای این بیماری به اثبات نرسیده است. یکی از این گزینه های درمانی، مهار کننده دارو های مهار کننده RNA پلیمراز ویروسی می باشند که برای درمان بیماری های ویروسی همچون آنفولانزا و ابولا طراحی شده اند. فاویپیراویر یکی از جدیدترین RNA پلیمراز است که در بیماری های زیادی مورد مطالعه قرار گرفته است.

فاویپیراویر با اصلاح ساختار شیمیایی یک آنالوگ پیرازین که در ابتدا با فعالیت چشم گیر ضد ویروس آنفولانزا در سلول های آزمایشگاهی همراه بود، کشف شد (19). فاویپیراویر یک مهار کننده انتخابی و قدرتمند RNA پلیمراز تعداد زیادی از زیر گروه ها و سویه های ویروس های آنفلوانزا می باشد که این حتی شامل مواردی می شود که نسبت به مهار کننده های نورآمینیداز و مهار کننده های M2 مقاوم هستند. فاویپیراویر فعالیت های ضد ویروسی را در برابر سایر ویروس های RNA دار نشان داده است (20). این داده ها به وضوح نشان می دهد که فاویپیراویر یک داروی امیدوارکننده برای درمان عفونت ها نه تنها ویروس آنفلوانزا بلکه طیف گسترده ای از ویروس های RNA است. از طرف دیگر، فاویپیراویر خطر تراتوژنیسیتی و سمیت جنینی را دارد (21).

در حال حاضر تعدادی کارآزمایی بالینی برای بررسی اثر این دارو در درمان بیماری COVID-19 در حال انجام می باشند و نتایج برخی از این مطالعات منتشر شده است. در یک مطالعه که در فوریه 2020 انجام گرفته است، مشخص شده میانگین مدت زمان لازم برای کاهش بار ویروس در تجویز داروی فاویپیراویر (4 روز) به صورت قابل توجهی نسبت به ترکیب داروهای لوپیناویر/ریتوناویر (11 روز) کمتر است (22). علاوه بر این، نتایج این مطالعه توسط یک کارآزمایی بالینی دیگر که در چین انجام شده است، تایید می شود، که در آن درمان با فاویپیراویر (4/71 درصد) به طور قابل توجهی میزان بهبود بیشتر در بیماران مبتلا به COVID-19 نسبت به امفنوویر[1] (9/55 درصد) نشان داده است (23). اگرچه در این کارآزمایی بالینی، میزان بهبود برای بیماران مبتلا به COVID-19 بحرانی مؤثر نبوده اند (5/5 درصد)، اما این نتایج اولیه منجر به شروع تعداد بیشتری کارآزمایی بالینی در ارتباط با این دارو ها در کشور های مختلف شد است (23).

در حالی که این دارو در آمریکا و بسیاری از کشور های اروپایی همچنان در درمان آنفولانزا تایید نشده است (12)، در سال 2014، ژاپن فاویپیراویر را برای درمان سویه های آنفلوانزا که نسبت به دارو های ضد ویروس فعلی مقاوم هستند، تصویب کرد. اگرچه اثر بخشی این دارو در درمان بیماری COVID-19 کماکان در دست بررسی می باشد، اما در 15 مارس 2020، این دارو برای درمان Covid-19 در چین تصویب شد (13). در 20 ژوئن 2020، این دارو در هند برای معالجه بیماران Covid-19 تحت نام تجاری Fabiflu تولید شده و مورد تصویب قرار گرفت (14, 15).

لوپیناویر یک مهار کننده پروتئاز ویروسی است، که ادعا می شود می تواند پروتئاز شبه ویروسی C3، ویروس هایی مانند MERS-CoV را مهار کند و آپوپتوز را در سلول های انسانی تعدیل کند (24, 25). کلترا یک برند دارویی است که داروی لوپیناویر تقویت شده با ریتوناویر می باشد و معمولاً به عنوان داروی ضد HIV/AIDS مورد استفاده قرار می گیرد. از دیگر دارو های ضد ویروسی می توان به ترکیب لوپیناویر/ریتوناویر اشاره کرد که تحت نام تجاری Kaletra فروخته می شود و یک داروی ترکیبی با دوز ثابت برای درمان و پیشگیری از HIV/AIDS است (16). به طور کلی شواهد نشان می دهد، ترکیب لوپیناویر با دوز کم ریتوناویر می تواند در درمان سایر ویروس ها خصوصا رتروویرس ها[2] می شود. این ترکیب در 15 سپتامبر 2000 در اروپا (17) در 19 مارس 2001 توسط سازمان غذا و دارو آمریکا برای درمان HIV/AIDS تصویب شده است (18).

لوپیناویر فعالیت ضد ویروس MERS-CoV با EC50 در حدود 0/8 میکرومولار را در شرایط آزمایشگاهی نشان می دهد. این غلظت زیر حداکثر غلظت پلاسمایی از دارو است که پس از مصرف یک دوز 500 میلی گرم خوراکی کلترا (400 میلی گرم لوپیناویر و 100 میلی گرم ریتوناویر) حاصل می شود (25).

در کارآزمایی های بالینی انجام شده، اثرات درمانی ترکیب لوپیناویر/ریتوناویر در بیماران مبتلا به SARS را مشخص شده است. در این مطالعه، در مقایسه با بیماران مبتلا به SARS که به مدت 21 روز ریباویرین دریافت کرده بودند، بیمارانی که علاوه بر ریباویرین، ترکیب لوپیناویر/ریتوناویر (400/100 میلی گرم 2 بار در روز) به مدت 14 روز دریافت کرده اند، دارای بیماری خفیف تر با شدت علائم کمتر همچون اسهال، عود تب، لنفوپنی، عفونت های بیمارستانی و بار ویروسی در ناحیه نازوفارنکس بودند (26). از طرفی اکثر بیماران یک دوره کوتاه با لوپیناویر/ریتوناویر را به خوبی تحمل کردند. اگرچه برخی از بیماران ناراحتی دستگاه گوارش را تجربه کرده بوند، افزایش آنزیم های کبدی، سردرد، تاری دید، کم خونی و برادی کاردی بدون علامت از دیگر عوارض با شیوع پایین می باشند (26). در گزارش یک بیمار مبتلا به MERS که لوپیناویر/ریتوناوویر در ترکیب با اینترفرون بتا-1a و ریباویرین دریافت کرده بود، پس از گذشت 2 روز از شروع درمان، وایرمی به صورت کامل بر ظرف گردید (27). همچنین در مطالعه انجام شد بر روی پریمات های مدل بیماری MERS مشخص شده است که تجویز درمان با لوپیناویر/ریتوناویر می تواند باعث بهبود مشخصه های بالینی، رادیولوژی و پاتولوژیک این بیماری شود (27).

در حال حاضر کارآزمایی های بالینی متععدی برای بررسی اثر این ترکیب دارویی در درمان بیماری COVID-19 در حال انجام است. در مطالعه ای که بر روی بیماران مبتلا به COVID-19 با علائم شدید انجام شد، مشخص گردید، تجویز ترکیب لوپیناویر/ریتوناویر (400/100 میلی گرم 2 بار در روز) به مدت 14 روز هیچ اثرات مفیدی معنی داری از این ترکیب را بر کاهش میزان مرگ و میر، کاهش مدت زمان بستری در بیمارستان، مدت زمان لازم برای کاهش بار ویروسی نشان نداده است (12). در مطالعه بالینی دیگر اثربخشی ترکیبی 14 روزه از لوپیناویر/ریتوناویر (400/100 میلی گرم 2 بار در روز)، ریباویرین 400 میلی گرم دوبار در روز و سه دوز 8 میلیون واحد بین المللی اینترفرون بتا-1b هر دو روز یکبار در مقایسه با لوپیناویر/ریتوناویر به تنهایی(گروه کنترل) مورد ارزیابی قرار گرفت. نتایج حاصل از این مطالعه نشان داد، مدت زمان لازم برای منفی شدن بار ویروسی در ناحیه نازوفارنکس در گروه درمان ترکیبی (7 روز) به صورت معنی داری کمتر از گروه کنترل (12 روز) بوده است. در حالی که شیوع عوارض ناشی از مداخله درمانی در بین دو گروه تغییر معنی داری نداشته است (28). در کارآزمایی بالینی دیگر که به منظور مقایسه اثرات درمانی ترکیبی از داروهای رمدسیویر (200 میلی گرم در روز اول و 100 میلی گرم در روزهای بعد برای 10 روز)، لوپیناویر/ریتوناویر در مقایسه با دارونما انجام شده است، مشخص گردید اثربخشی در گروه دریافت کننده درمان در مقایسه با گروه دریافت کننده دارو نما تغییر معنی داری با یکدیگر ندارند (29).

**اهداف كلي طرح:**

- بررسی اثربخشی و ایمنی فاویپیراویر و اینترفرون بتا در مقایسه با لوپیناویر/ریتوناویر و اینترفرون بتا در بیماران مبتلا به COVID-19

**اهداف اختصاصی طرح :**

- ب بررسی اثربخشی فاویپیراویر و اینترفرون بتا-1a بر کاهش بار ویروسی در بیماران مبتلا به COVID-19
- بررسی اثربخشی فاویپیراویر و اینترفرون بتا-1a بر بهبود علائم بالینی در بیماران مبتلا به COVID-19
- بررسی سمیت و عوارض جانبی فاویپیراویر و اینترفرون بتا-1a در بیماران مبتلا به COVID-19
- بررسی اثربخشی لوپیناویر/ریتوناویر و اینترفرون بتا-1a بر کاهش بار ویروسی در بیماران مبتلا به COVID-19
- بررسی اثربخشی لوپیناویر/ریتوناویر و اینترفرون بتا-1a بر بهبود علائم در بیماران مبتلا به COVID-19
- بررسی سمیت و عوارض جانبی لوپیناویر/ریتوناویر و اینترفرون بتا-1a در بیماران مبتلا به COVID-19

**اهداف كاربردي طرح :**

در صورت مشاهده اثرات مثبت از فاویپیراویر و اینترفرون بتا و یا لوپیناویر/ریتوناویر و اینترفرون بتا، این رژیم های درمانی می توانند به بهبود کارایی رژیم های درمانی کمک کنند و در صورت حصول پاسخ دهی بهتر نسبت به درمان استاندارد می توان در آینده این رژیم ها به عنوان درمان های استاندارد جهت بهبود سریع تر علائم بالینی و یافته های آزمایشگاهی بیماران استفاده شوند.

- انتظار می رود که این مطالعه به تدوین پروتکل درمانی در بیماری COVID-19 کمک کند.
- انتظار می رود که این مطالعه منجر به کاهش مشکلات درمانی دربیماری COVID-19 شود.
- انتظار می رود که نتایج این تحقیق منجر به راه کارهایی جهت کنترل بیماری COVID-19 در کشور شود.
- انتظار می رود که نتایج این تحقیق از طریق کاهش مشکلات بالینی منجر به کاهش هزینه های درمانی در بیماران COVID-19 شود.

**فرضيات يا سوالات پژوهش (باتوجه به اهداف طرح) :**

- تجویز فاویپیراویر و اینترفرون بتا-1a باعث کاهش بار ویروسی در بیماران مبتلا به COVID-19 می شود.
- تجویز فاویپیراویر و اینترفرون بتا-1a باعث بهبود علائم بالینی در بیماران مبتلا به COVID-19 می شود.
- عوارض جانبی فاویپیراویر و اینترفرون بتا-1a در بیماران مبتلا به COVID-19 قابل تحمل می باشد.
- تجویز لوپیناویر/ریتوناویر و اینترفرون بتا-1a باعث کاهش بار ویروسی در بیماران مبتلا به COVID-19 می شود.
- تجویز لوپیناویر/ریتوناویر و اینترفرون بتا-1a باعث بهبود علائم در بیماران مبتلا به COVID-19 می شود.
- عوارض جانبی لوپیناویر/ریتوناویر و اینترفرون بتا-1a در بیماران مبتلا به COVID-19 قابل تحمل می باشند..

**روش اجرای طرح**

**طراحی مطالعه و شرکت کنندگان**

این مطالعه در قالب یک کارآزمایی بالینی تصادفی با برچسب باز[1] بر روی 30 بیمار مبتلا به بیماری COVID-19 بستری شده در بخش سندرم حاد تنفسی بیمارستان شهید محمدی شهرستان بندرعباس، ایران طراحی شده است. بیماران از 25 ژوئیه 2020 به صورت آینده نگر وارد مطالعه و پیگیری خواهند شد. با توجه به نسبت 1:1 بین گروه های مورد مطالعه (گروه دریافت کننده فاویپیراویر و گروه دریافت کننده لوپیناویر/ریتوناویر)، بیماران به صورت تصادفی وارد هر یک از بازو های مداخله می شوند.

در شروع مطالعه بیماران بر اساس علائم بالینی و یافته های پاراکلینیکی در گروه های خفیف، متوسط، شدید و بحرانی تقسیم بندی می شوند (جدول 1). معیارهای ورود بیماران شامل؛ (1) سن 18 سال یا بالاتر، (2) رضایت آگاهانه و داوطلبانه، (3) تشخیص قطعی بیماری COVID-19 از طریق تست PCR[2]، (4) علائم اولیه بیماری با شدت متوسط و یا شدید که در بیمارستان بستری شده اند، خواهند بود. تمامی بیماران با سابقه ای از (1) هپاتیت مزمن، سیروز کبدی، بیماری های کلستاتیک کبد و التهاب کیسه صفرا، (2) نارسایی های حاد و مزمن کلیوی، (3) زخم های گوارشی، صرع، بیماری های قلبی، بیماری های روانی کنترل نشده، (4) حساسیت به دارو های مورد مطالعه، (5) زنان در دوران بارداری و شیردهی، (6) مصرف سایر داروهای ضد ویروس، داروهایی مهار کننده سیستم آنزیمی سیتوکروم اکسیداز کبدی و سرکوبگر های ایمنی، (7) مصرف الکل، (8) متابولیسم غیر نرمال اسید اوریک، (21) عفونت های همزمان باکتریال سیستم تنفسی و (22) ابتلا به بیماری های مزمن سخت مانند سرطان و ایدز از مطالعه خارج می شوند. در ابتدای کارآزمایی، مشخصات عمومی، مشخصات دموگرافیک و سوابق پزشکی بیماران با استفاده از پرسشنامه استاندارد طراحی شده در این زمینه جمع آوری می شود. پس از ارائه توضیحات کافی و کسب رضایت آگاهانه کتبی از جانب بیمار یا بستگان درجه یک (در بیماران با سطح هوشیاری پایین یا زوال عقل)، بیماران با استفاده از روش تصادفی سازی بلوک ها دو گروه مساوی (گروه مداخله و گروه کنترل) تقسیم می شوند.

**گروه های مداخله**

گروه A شامل بیمارانی هستند که درمان آنها از داروی فاویپیراویر استفاده می شود. این دارو به صورت 1600 میلی گرم (8 قرص 200 میلی گرمی) در شروع و 600 میلی گرم (3 قرص 200 میلی گرمی) دو بار در روز در ادامه (روزهای 2 تا 5) تجویز می شود. گروه B شامل بیمارانی هستند که داروی لوپیناویر/ریتوناویر (200/50 میلی گرم دو بار در روز) برای 7 روز دریافت می کنند. هر دو گروه A و B علاوه بر درمان فوق 5 دوز تزریق زیرجلدی 44 میکرو گرم اینترفرون بتا-1a به صورت یک روز درمیان دریافت خواهند کرد.

در این مطالعه از داروی فاویپیراویر (قرص های 200 میلی گرمی، شرکت Zhejiang Hisun، چین)، کلترا (قرص های ترکیبی حاوی دارو های لوپیناویر 200 میلی گرم و ریتوناویر 50 میلی گرم، شرکت باختر بیوشیمی، ایران) و رسیژن (آمپول های از پیش پر شده حاوی اینترفرون بتا-1a به میزان 44 میکرو گرم معادل 12000000 واحد بین المللی، شرکت سیناژن، ایران) استفاده خواهد شد.

**بررسی پیامد ها**

پیامد های اولیه این مطالعه شامل کاهش بار ویروسی در تست PCR در انتهای مطالعه (روز هفتم و یا زمان ترخیص) و میزان بهبود علائم بالینی در طی دوره مداخله نظر گرفته می شود. بهبود علائم بالینی به عنوان بهبود مداوم (بیشتر از 72 ساعت) درجه حرارت بدن، فرکانس تنفس و میزان اشباع اکسیژن خون پس از شروع درمان می باشد که با معیارهای کمی که در ادامه آمده است تعریف می شود: دمای دهانی ≥ 6/36 درجه سانتیگراد؛ فرکانس تنفسی ≥24 بار در دقیقه و اشباع اکسیژن ≤98 درصد بدون تنفس مکانیکی. علاوه بر این، نیاز به اکسیژن درمانی و تهویه با فشار مثبت غیر تهاجمی در فالوآپ های روزانه به همراه علائم بالینی دیگر علائم بالینی دیگر همچون سرفه، درد عضلانی، سردرد، تنگی نفس، ضعف و بی حالی، کاهش حس بویایی و چشایی، اسهال، اسپاسم شکمی، تهوع و استفراغ به صورت کیفی ثبت می شوند. اندازه گیری مکرر حداقل برای دو بار در هر فالوآپ انجام می شود.

پیامد های ثانویه شامل مدت زمان بستری بودن در بیمارستان، نیاز به بستری شدن در بخش مراقبت های ویژه و میزان بهبود پارامتر های بیوشیمیایی بیماران می باشند. انجام آزمایش های CBC[3]، شمارش تفریقی سلول های سفید، میزان فریتین، CRP[4]، LDH[5]، ESR[6]، کراتینین و نیتروژن اوره سرم در ابتدای مطالعه و انتهای مطالعه (روز 7 مطالعه و یا زمان ترخیص) بررسی خواهد شد. همچنین عوارض جانبی ناشی از دارو های مورد مطالعه (به ویژه عوارضی همچون افزایش فشار خون، تهوع و استفراغ، اسهال، اسپاسم های گوارشی، ضعف و بیحالی، سردرد، و بثورات جلدی)، فراوانی عوارض جانبی احتمالی ناشی از مداخله و فراوانی انصراف از مطالعه به علت عوارض جانبی در گروه های مورد مطالعه به صورت روزانه ثبت می شوند.

[1] Open-label randomized clinical trial

[2] Polymerase Chain Reaction

[3] Complete blood count

[4] C-reactive protein

[5] Lactate dehydrogenase

[6] Erythrocyte sedimentation rate

**روش محاسبه حجم نمونه**

با استفاده از مطالعات مشابه انجام شده در گروه های چند دارویی میزان بهبودی بالینی مورد انتظار در روز پنجم از گروه مورد مطالعه 83 درصد، بهبودی بالینی گروه کنترل 35 درصد، α = 0.05 ، β = 0.10، قدرت = 90/0 در نظر گرفته شد. با توجه به توزیع 1: 1 بین گروه های مورد مطالعه، حجم نمونه آماری 24 شرکت کننده در هر گروه محاسبه گردید. حجم نمونه با توجه به عواملی مانند ریختن نمونه ها حدود 20 درصد بیشتر در نظر گرفته می شود. بنابراین این کارآزمایی شامل 60 نفر (30 نفر در هر گروه) می باشد.


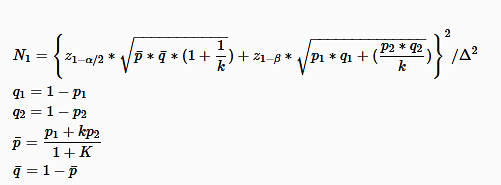


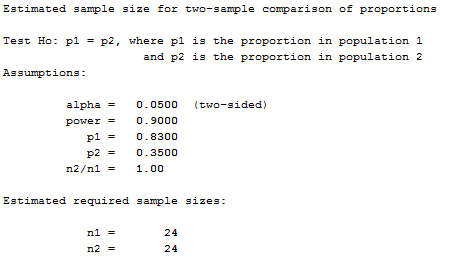


**روش تصادفی سازی بلوک**

در این روش تعداد افراد در هر یک از گروه های مطالعه در طول درمان با یکدیگر برابر است. با توجه به تعداد 60 نفر افراد شرکت کننده (30 نفر در هر گروه) و مدت زمان تقریبی 10 هفته برای تکمیل ورود افراد به مطالعه، از 10 بلوک 6 تایی (در صورت وجود بیمار به تعداد کافی از 5 بلوک 12 تایی) استفاده خواهد شد. روش کار در این نوع از تصادفی سازی شبیه به روش تصادفی سازی ساده است، تنها تعداد افراد در طول دوره درمان در دو گروه درمان یکسان می باشد. تنها ایراد این روش مشخص شدن آخرین گروه در هر بلوک می باشد.

برای تحلیل آماری از نرم افزار SPSS ورژن 0/18 استفاده می شود. برای مقایسه شاخص های اصلی اثر بخشی (کاشه بار ویروسی و میزان بهبود علائم بالینی) به عنوان پیامد اولیه و پیامد های ثانویه بین گروه های مورد مطالعه، از آزمون t برای متغیرهای کمی پیوسته و یا آزمون Wilcoxon (در صورت عدم استفاده از آزمون t) برای متغییر های کمی گسسته رتبه ای استفاده می شود. توصیف آماری متغیرهای کیفی به صورت فراوانی یا درصد مشاهده خواهد بود و برای مقایسه بین گروه ها از آزمون های Chi-square یا Fisher’s exact استفاده خواهد شد. برای کلیه آزمون های آماری، P <0.05 (دو طرفه) از نظر آماری معنی دار در نظر گرفته می شود.

**ملاحظات اخلاقی**

جهت شرکت در این مطالعه از تمامی بیماران رضایت آگاهانه اخذ خواهد شد. اطلاعات مربوطه بصورت محرمانه حفظ خواهد شد. هیچ یک از مشخصات فردی افراد شرکت کننده از جمله اسم و فامیل آنها وارد کامپیوتر نخواهد شد و به تمامی افراد کد پروژه داده شده و آنالیز بر اساس آن انجام خواهد شد. اطلاعات اولیه در فایل های قفل دار و نزد مجری اصلی تا اتمام پروژه و انتشار مقالات باقی خواهد ماند. در هر مرحله از طرح بیماران می توانند بنا به تمایل شخصی از طرح خارج شوند.

**محدودیت‌های اجرایی طرح و روش رفع آن ها**

عدم همکاری بیماران که امید است با توضیحات کافی برای آن ها این مشکل رفع شود و قبل از شروع به بیماران در مورد عوارض جانبی بیماری و دارو توضیح داده می شود. از آنها قبل از شروع درمان رضایت نامه کتبی دریافت می شود. همچنین این پژوهش از نظر اخلاق پزشکی مورد تایید است. یکی از محدودیت های مهم دیگر در این طرح کمبود منابع دارویی خصوصا راجع به داروهای فاویپیراویر و کلترا می باشد که امید است با توجه به مکاتبات لازم با معاونت های پژوهشی و غذا و دارو دانشگاه علوم پزشکی هعرمزگان و پشتیبانی ریاست دانشگاه این مشکل رفع گردد.

**References**

1.            Lai C-C, Shih T-P, Ko W-C, Tang H-J, Hsueh P-R. Severe acute respiratory syndrome coronavirus 2 (SARS-CoV-2) and corona virus disease-2019 (COVID-19): the epidemic and the challenges. International journal of antimicrobial agents. 2020:105924.

2.            Wang L-s, Wang Y-r, Ye D-w, Liu Q-q. A review of the 2019 Novel Coronavirus (COVID-19) based on current evidence. International Journal of Antimicrobial Agents. 2020:105948.

3.            Organization WH. WHO Director-General's opening remarks at the media briefing on COVID-19-11 March 2020. Geneva, Switzerland. 2020.

4.            Kailas Khandu Sanap D, Sanap AK. What We Know So Far About New Coronavirus (COVID-19). Sustainable Humanosphere. 2020;16(1):1470-6.

5.            Magdi H. COVID-19 Coronavirus Disease. 2020.

6.            Hopkins C, Kumar N. Loss of sense of smell as marker of COVID-19 infection. ENT UK at The Royal College of Surgeons of England. 2020.

7.            Velavan TP, Meyer CG. The COVID-19 epidemic. Trop Med Int Health. 2020;25(3):278-80.

8.            Hui DS, Azhar EI, Madani TA, Ntoumi F, Kock R, Dar O, et al. The continuing 2019-nCoV epidemic threat of novel coronaviruses to global health—The latest 2019 novel coronavirus outbreak in Wuhan, China. International Journal of Infectious Diseases. 2020;91:264.

9.            Wu Z, McGoogan JM. Characteristics of and important lessons from the coronavirus disease 2019 (COVID-19) outbreak in China: summary of a report of 72 314 cases from the Chinese Center for Disease Control and Prevention. Jama. 2020.

10.          Colson P, Rolain J-M, Lagier J-C, Brouqui P, Raoult D. Chloroquine and hydroxychloroquine as available weapons to fight COVID-19. Int J Antimicrob Agents. 2020;105932(10.1016).

11.          Colson P, Rolain J-M, Raoult D. Chloroquine for the 2019 novel coronavirus. Int J Antimicrob Agents. 2020.

12.          Cao B, Wang Y, Wen D, Liu W, Wang J, Fan G, et al. A Trial of Lopinavir-Ritonavir in Adults Hospitalized with Severe Covid-19. The New England journal of medicine. 2020;382(19):1787-99.

13.          Yangfei Z. 'Potential coronavirus drug approved for marketing'. Chinadaily.com.cn. Retrieved 2020-03-21.

14.          DelhiJune 20, India Today Web Desk New; June 20, 2020UPDATED:; Ist, 2020 22:30. 'Glenmark's FabiFlu approved for coronavirus treatment in India, costs Rs 103 per tablet.

15.          'Glenmark Launches COVID-19 Drug Favipiravir At Rs 103 Per Tablet'. NDTV.com. Retrieved 2020-06-22.

16.          'Lopinavir and Ritonavir'. The American Society of Health-System Pharmacists. Archived from the original on 20 December 2016. Retrieved 28 November 2016.

17.          'Kaletra EPAR'. European Medicines Agency (EMA). Retrieved 18 February 2020.

18.          'Drug Approval Package: Kaletra (Lopinavir/Ritonavir) NDA #21-226 & 21-251'. U.S. Food and Drug Administration (FDA). 20 November 2001. Retrieved 18 March 2020.

19.          Furuta Y, Takahashi K, Kuno-Maekawa M, Sangawa H, Uehara S, Kozaki K, et al. Mechanism of action of T-705 against influenza virus. Antimicrobial agents and chemotherapy. 2005;49(3):981-6.

20.          Furuta Y, Gowen BB, Takahashi K, Shiraki K, Smee DF, Barnard DL. Favipiravir (T-705), a novel viral RNA polymerase inhibitor. Antiviral research. 2013;100(2):446-54.

21.          Nagata T, Lefor AK, Hasegawa M, Ishii M. Favipiravir: a new medication for the Ebola virus disease pandemic. Disaster medicine and public health preparedness. 2015;9(1):79-81.

22.          Xuan Y. Initial clinical results announced for favipiravir treatment of novel coronavirus pneumonia-viral clearance in four days. Biodiscover. 2020.

23.          Chen C, Huang J, Cheng Z, Wu J, Chen S, Zhang Y, et al. Favipiravir versus arbidol for COVID-19: a randomized clinical trial. MedRxiv. 2020.

24.          de Wilde AH, Jochmans D, Posthuma CC, Zevenhoven-Dobbe JC, van Nieuwkoop S, Bestebroer TM, et al. Screening of an FDA-approved compound library identifies four small-molecule inhibitors of Middle East respiratory syndrome coronavirus replication in cell culture. Antimicrobial agents and chemotherapy. 2014;58(8):4875-84.

25.          Rizza SA, Badley AD. HIV protease inhibitors impact on apoptosis. Medicinal Chemistry. 2008;4(1):75-9.

26.          Chu C, Cheng V, Hung I, Wong M, Chan K, Chan K, et al. Role of lopinavir/ritonavir in the treatment of SARS: initial virological and clinical findings. Thorax. 2004;59(3):252-6.

27.          Spanakis N, Tsiodras S, Haagmans BL, Raj VS, Pontikis K, Koutsoukou A, et al. Virological and serological analysis of a recent Middle East respiratory syndrome coronavirus infection case on a triple combination antiviral regimen. International journal of antimicrobial agents. 2014;44(6):528-32.

28.          Hung IF-N, Lung K-C, Tso EY-K, Liu R, Chung TW-H, Chu M-Y, et al. Triple combination of interferon beta-1b, lopinavir–ritonavir, and ribavirin in the treatment of patients admitted to hospital with COVID-19: an open-label, randomised, phase 2 trial. The Lancet. 2020;395(10238):1695-704.

29.          Wang Y, Zhang D, Du G, Du R, Zhao J, Jin Y, et al. Remdesivir in adults with severe COVID-19: a randomised, double-blind, placebo-controlled, multicentre trial. Lancet (London, England). 2020;395(10236):1569-78.
